# PILOT OUTCOMES OF A COMMUNITY-BASED FALLS PREVENTION PROGRAM TARGETING OLDER ARAB AMERICANS

**DOI:** 10.1093/geroni/igz038.3185

**Published:** 2019-11-08

**Authors:** Lan Yao, Suha Kridli, Amne Talab

**Affiliations:** 1 Oakland University, Rochester, Michigan, United States; 2 Arab Community Center for Economic and Social Services, Dearborn, Michigan, United States

## Abstract

Arab American (AA) is the 3rd largest ethnic population in the state of Michigan. Previous studies found that Michigan Arabs were less healthy than the general population in Michigan. Older AAs have higher mortality risk than non-Arab and non-Hispanic Whites, particularly due to chronic diseases. Community-based programs are an effective approach to prevent disease and injury, improve health, and enhance quality of life. While evidence for functional gains resulting from Tai Chi exercise is accumulating, there is little research and support for its feasibility and effectiveness that target older AAs, who are not culturally related to Tai Chi. Participants in this report were 8 older female AAs (mean age 62.4±3.2, range 58-66) who sought services at a not-for-profit Arab Community Center, which aims to enable and empower residents and communities to lead informed, productive and culturally sensitive lives. A certified Tai Chi instructor led the classes using Yang-style Tai Chi moves. The participants completed a 12-week twice-weekly 1-hour Tai Chi classes and post-program focus group discussion, held in a classroom of the Community Center. Post-intervention improvement in timed up & go test (p>.05), comfortable & fast gait speed (p>.05), unipedal stance time (p>.05) were observed. Themes identified from focus group supported Tai Chi’s benefits in balance and salient mental health benefits. The pilot data provides preliminary evidence for therapeutic gains resulting from Tai Chi practices. Agencies serving older AAs play important roles by creating and promoting evidence-based health promotion practices to address the growing needs among older adults.

